# Species composition and population dynamics of *Aedes* mosquitoes, potential vectors of arboviruses, at the container terminal of the autonomous port of Abidjan, Côte d’Ivoire

**DOI:** 10.1051/parasite/2013013

**Published:** 2013-04-09

**Authors:** Yao Lucien Konan, Zanakoungo Ibrahim Coulibaly, Atiuomounan Blaise Koné, Kouadio Daniel Ekra, Julien Marie-Christian Doannio, Mirielle Dosso, Paul Odéhouri-Koudou

**Affiliations:** 1 Vector Control Department, National Institute of Public Hygiene PO Box V 14 Abidjan Côte d’Ivoire; 2 Laboratory of Environmental Sciences, UTR of Science and Environmental Management, University of Abobo-Adjamé PO Box 801 Abidjan 02 Côte d’Ivoire; 3 National Institute of Public Hygiene PO Box V 14 Abidjan Côte d’Ivoire; 4 Research Unit “Vector-Borne and Neglected Tropical Diseases Research Unit,” National Institute of Public Health PO Box V 47 Abidjan Côte d’Ivoire; 5 Entomology and Herpetology Research Unit, Pasteur Institute of Côte d’Ivoire Abidjan PO Box 490 Abidjan 01 Côte d’Ivoire; 6 Pasteur Institute of Côte d’Ivoire PO Box 490 Abidjan 01 Côte d’Ivoire

**Keywords:** Entomological survey, *Aedes* species, Container terminal, Côte d’Ivoire, Ivory Coast

## Abstract

An entomological survey of *Aedes* mosquitoes was initiated at the container terminal of the Autonomous Port of Abidjan in 2009 following the occurrence of two outbreaks of yellow fever in less than 10 years and dengue cases reported in 2008 among expatriates returning from Côte d’Ivoire (Ivory Coast). It was based on regular monitoring of ovitraps from July 2009 to June 2010. A total of 4,739 adult mosquitoes represented by four species of *Aedes* (97% of total) and one unexpected species of *Culex* (3%) were obtained. *Aedes aegypti* was dominant with 98% of total *Aedes* (*n* = 4,594). Its density variation was closely related to the amount of rainfall. The other species of *Aedes* were collected in the second half of the major rainy season including *Ae. albopictus* (1.17% of *Aed*es) and *Ae. angustus* (0.13%) whose presence was discovered for the first time in Côte d’Ivoire.

## Abstract (fr)

Une enquête entomologique portant sur les moustiques du genre *Aedes* a été initiée au terminal à conteneurs du Port Autonome d’Abidjan en 2009 suite à la survenue de deux épidémies de fièvre jaune en moins de 10 ans et des nombreux cas de dengue rapportés en 2008 chez des expatriés de retour d’un séjour en Côte d’Ivoire. Elle a été basée sur le suivi régulier des pondoirs pièges de juillet 2009 à juin 2010. Un total de 4,739 moustiques adultes répartis entre 4 espèces d’*Aedes* (97 % du total) et une espèce de *Culex* (3 %) inattendue a été obtenu. *Aedes aegypti* était prédominant avec 98 % du total des *Aedes* (*n* = 4,594). La variation de sa densité est étroitement liée à la quantité de pluie. Les autres espèces d’*Aedes* ont été récoltées dans la seconde moitié de la grande saison des pluies, dont *Ae. albopictus* (1.17 % des *Aedes*) et *Ae. angustus* (0.13 %) récoltés pour la première fois en Côte d’Ivoire.

## Introduction

Diseases which have been known for a long time such as yellow fever and dengue are currently reappearing in many parts of the world due to economic and social deterioration and public health systems in these countries [16]. Despite the existence of an effective vaccine for many years, yellow fever continues to be an endemic disease and an important threat in Africa and South America. The inadequate vaccine coverage and population movements explain the continual occurrence of epidemics. The WHO estimates that 200,000 cases of yellow fever occur each year worldwide claiming 30,000 deaths [19]. More than 90% of all cases are in Africa, where more than 500 million people live in the risk zone situated between latitudes 15° N and 15° S. Yellow fever is also a significant risk for more than 3 million travelers who enter the affected areas each year [20]. Dengue fever is one of the most important emerging infectious diseases worldwide with at least 20 million cases per year claiming 25,000 lives [7]. Approximately three billion people are under the risk of infection. The annual incidence of dengue has been multiplied by 30 during the last 50 years with increasing number of cases which has become exponential in the last 15 years [27]. This pathology is now part of the diseases of traveler’s returning from the tropics [9]. There are four different serotypes of dengue virus. Infection with one serotype confers protective immunity against this serotype but not against others. A second infection by another serotype causes a higher risk of dengue hemorrhagic fever, which is a more severe form of the disease [7]. Unlike yellow fever, there is no vaccine for dengue, but early detection and access to appropriate medical care help to reduce mortality rates below 1% [22]. Yellow fever and dengue are viral infectious diseases transmitted by mosquitoes of the genus *Aedes*. These mosquitoes have the particularity of eggs that resist drought, a condition that allows them to survive prolonged journey by sea, air, and land. This factor has enabled them to spread worldwide [5].

Côte d’Ivoire is an endemic yellow fever country which recently experienced a resurgence of this disease after nearly two decades of relative calm following the introduction of yellow fever vaccine in the nationwide vaccination program in 1983. Between 2001 and 2008, three outbreaks occurred in Abidjan, the economic capital of the country. In the 2001 yellow fever outbreak, 16 out of the 73 suspected cases were laboratory-confirmed with one fatal case [1]. Seven years later, another yellow fever outbreak occurred with 15 cases confirmed by the Pasteur Institute of Côte d’Ivoire; the first five of these were counter-confirmed by the regional reference laboratory of the Pasteur Institute of Dakar [17, 21]. Almost at the same period, an epidemic of Middle Eastern type 3 dengue was detected following an international alert launched after a positive diagnosis of dengue among two tourists returning from Côte d’Ivoire [21]. These two epidemics of yellow fever occurring in less than 10 years and dengue cases reported in 2008 among expatriates returning from Côte d’Ivoire obliged the Ministry of Health and Public Hygiene at the time to strengthen its warning system. It is in this context that surveillance of *Aedes* mosquitoes which are potential arbovirus vectors was initiated at strategic points in the capital city including the container terminal of the Autonomous Port of Abidjan. This entomological surveillance aimed at following up the species composition of populations of *Aedes* mosquitoes and subsequently the presence of arboviruses among the vectors identified. This article presents results of the monitoring carried out from July 2009 to June 2010 at the container terminal of the Autonomous Port of Abidjan.

## Materials and methods

### Study site

The city of Abidjan, with a population of over 6 million, is located in the southern part of Côte d’Ivoire (5°19′ N, 4°01′ W). The city comprises 10 municipalities and a national park (called Banco National Park) which spreads over 3,750 hectares located in the northern part of the municipality of Attécoubé. The climate is tropical with two rainy seasons separated from each other by a dry season. The major rainy season is from April to July and the shorter from October to November. The major dry season starts from December to March and a short one occurs from August to September [4, 12]. The annual average rainfall is between 1,300 and 1,600 mm. According to SODEXAM (Ivorian firm for the Development and Exploitation of Meteorological Parameters), the mean annual temperature is around 26.5 °C. The annual relative humidity is between 78% and 88%.

The container terminal of the autonomous Port of Abidjan, located in the municipality of Port Bouet, was one of the sites chosen to conduct this pilot surveillance of potential vectors of arboviruses in Abidjan. Covering an area of 30 hectares, it is located at the edge of the lagoon Ebrié which communicates with the Atlantic Ocean through the Vridi canal built in 1935. The principal activities that are undertaken at the container terminal are: reception and delivery of arriving containers, preparation of outgoing containers; checking storage and monitoring of containers at the container park; loading and unloading of container ships, including shifting and transhipment of containers.

### Mosquito collection

Ovitraps method [8] was used to collect *Aedes* eggs. Thirty-nine standard WHO ovitraps were placed in six different points, spread over the container terminal installation, according to the field plan and the agreement of the operating company. These are: staff parking here referred to as “Parking” (6 ovitraps), the customs here called as “Customs” (5 ovitraps), storage site of petroleum products here designated as “Dangerous site” (9 ovitraps), empty containers parking here nominated “Empty park” (10 ovitraps), maintenance workshop here referred to as “Workshop” (5 ovitraps), and cold containers park or “Cold park” (4 ovitraps). These ovitraps are black empty cans in which small wooden paddles are immersed. These were installed at 1.5 m above the ground. Paddles were collected every 10 days. At each harvest, water contained in cans was transferred and renewed. These different harvests were collected in different bags labeled according to each collection point and carried to the laboratory. At the laboratory, larvae and pupae in the collected water of the traps were counted. The larvae were transferred to basins containing dechlorinated water. Cat food (Purina, Friskies) was supplied to feed the larvae. Pupae were transferred to plastic cups (25 cc) and transferred to square metal cages (30 cm^3^) for the adults’ emergence. The resulting adults were identified using the morphological identification keys [13] and morphological descriptions of African *Aedes* species [15]. The number of adults was recorded for each harvest point. Paddles were dried on a table covered with mosquito nets to prevent laying of external mosquitoes. After drying, the paddles were immersed for 2 days in dechlorinated water with yeast tablets to induce larval hatching. They were then removed, leaving the hatched larvae in the water, and allowed to dry for 5 days before being again immersed for 2 days. This process was repeated three times. Larvae obtained from hatched eggs were also counted, reared, and treated in the same way as above.

Data were analyzed using Statistica Software version 7.1. A Generalized Linear Model (GLM) framework [6] was used to compare larvae mortality during rearing using binomial error. The average number of *Ae. aegypti* obtained was compared using a procedure repeated measures analysis with a general linear model in order to take into account the possible effects of collection sites and rainfall on adult density.

## Results

From July 2009 to June 2010, a total of 4,739 adult mosquitoes (48.7% females, 51.3% males) were obtained at the Container Terminal of the Autonomous Port of Abidjan (Table 1). This population was dominated by *Aedes* species which constituted 96.94% of the emergences. *Aedes* mosquitoes were represented by four species led by *Aedes aegypti* (Linnaeus, 1762) with 98.39% of all *Aedes* spp. collected (*n* = 4594) followed respectively by *Aedes albopictus* Skuse, 1894 with 1.17%, *Aedes apicoargenteus* (Theobald, 1909) with 0.30%, and *Aedes angustus* Edwards, 1935 with 0.13%. One unexpected species of *Culex* representing 3.06% of the mosquitoes collected was obtained in the water contained in the traps.

**Table 1.** Species composition of mosquitoes collected using ovitraps at the container terminal of the port of Abidjan (Côte d’Ivoire) from July 2009 to June 2010.

Species	Females	%	Males	%	Total	%
*Aedes aegypti*	2,190	46.21	2,330	49.17	4,520	95.38
*Aedes albopictus*	33	0.7	21	0.44	54	1.14
*Aedes apicoargenteus*	8	0.17	6	0.13	14	0.3
*Aedes angustus*	4	0.08	2	0.04	6	0.13
*Culex nebulosus*	73	1.54	72	1.52	145	3.06
Total	2,308	48.7	2431	51.3	4,739	100

### Relative abundance of *Aede*s species collected

The average number of *Aedes* was 4.6 specimens per paddle (S/Pl) for the study period with a minimum of 2.8 S/Pl collected at the Workshop and a maximum of 7.6 S/Pl at the Cold park (Table 2). Comparison of larvae mortality during their rearing showed no significant differences thus making possible the comparison of adults obtained between sites (*p* = 0.730). The average number of *Aedes* per paddle differed significantly according to collection points (*p* = 0.006), with no interaction with the rainy season (*p* = 0.294). However, the average number of *Aedes* collected at Parking is comparable with that of Customs (*p* = 0.212). Similarly the average number at Parking is comparable with that of Dangerous site (*p* = 0.111), Parking is comparable with Empty park (*p* = 0.074), and finally Customs with Dangerous site (*p* = 0.728). The collection sites can thus be classified into four groups (Table 3).

**Table 2.** Average number of *Aedes* mosquitoes collected using ovitraps in different points of the container terminal port of Abidjan (Côte d’Ivoire) from July 2009 to June 2010. For each number: average number of specimens per paddle (total number of specimens).

	Parking	Customs	Dangerous site	Empty park	Workshop	Cold park	Total
Number of paddles collected	148	139	237	268	126	76	994
*Aedes aegypti*	4.62 (684)	3.85 (536)	3.74 (888)	5.56 (1,491)	2.64 (346)	7.56 (575)	4.54 (4,520)
*Aedes albopictus*	0 (0)	0 (0)	0 (0)	0.17 (47)	0.03 (4)	0.04 (3)	0.05 (54)
*Aedes apicoargenteus*	0 (0)	0.05 (8)	0 (0)	0 (1)	0 (0)	0.07 (5)	0.01 (14)
*Aedes angustus*	0 (0)	0 (0)	0 (0)	0 (0)	0.05 (6)	0 (0)	0.00 (6)
TOTAL	4.62 (684)	3.91 (544)	3.74 (888)	5.74 (1,539)	2.82 (356)	7.66 (583)	4.62 (4,594)

**Table 3.** Classification of collection sites according to the average number of adult mosquitoes obtained at the container terminal of the port of Abidjan (Côte d'Ivoire)

Collection sites	Mean	Groups	Groups	Groups	Groups
Workshop	2.70 ± 1.14	A			
Dangerous site	3.74 ± 1.05		B		
Customs	3.92 ± 1.04		B		
Parking	4.62 ± 1.03		B	C	
Empty park	5.74 ± 1.36			C	
Cold park	7.66 ± 1.61				D

The number of *Aedes* species varied from one collection point to another but with the presence of *Ae. aegypti* at each collection point. At Parking and at Dangerous site only *Ae. aegypti* was collected. At Customs, *Ae. apicoargenteus* was collected with *Ae. aegypti*. At the other three points, three species were collected, namely *Ae. aegypti*, *Ae. albopictus*, and *Ae. apicoargenteus* at Empty park and at Cold park, and *Ae. aegypti*, *Ae. albopictus*, and *Ae. angustus* at the Workshop (Table 2).

### Variation of *Aedes* density

**Fig. 1.**
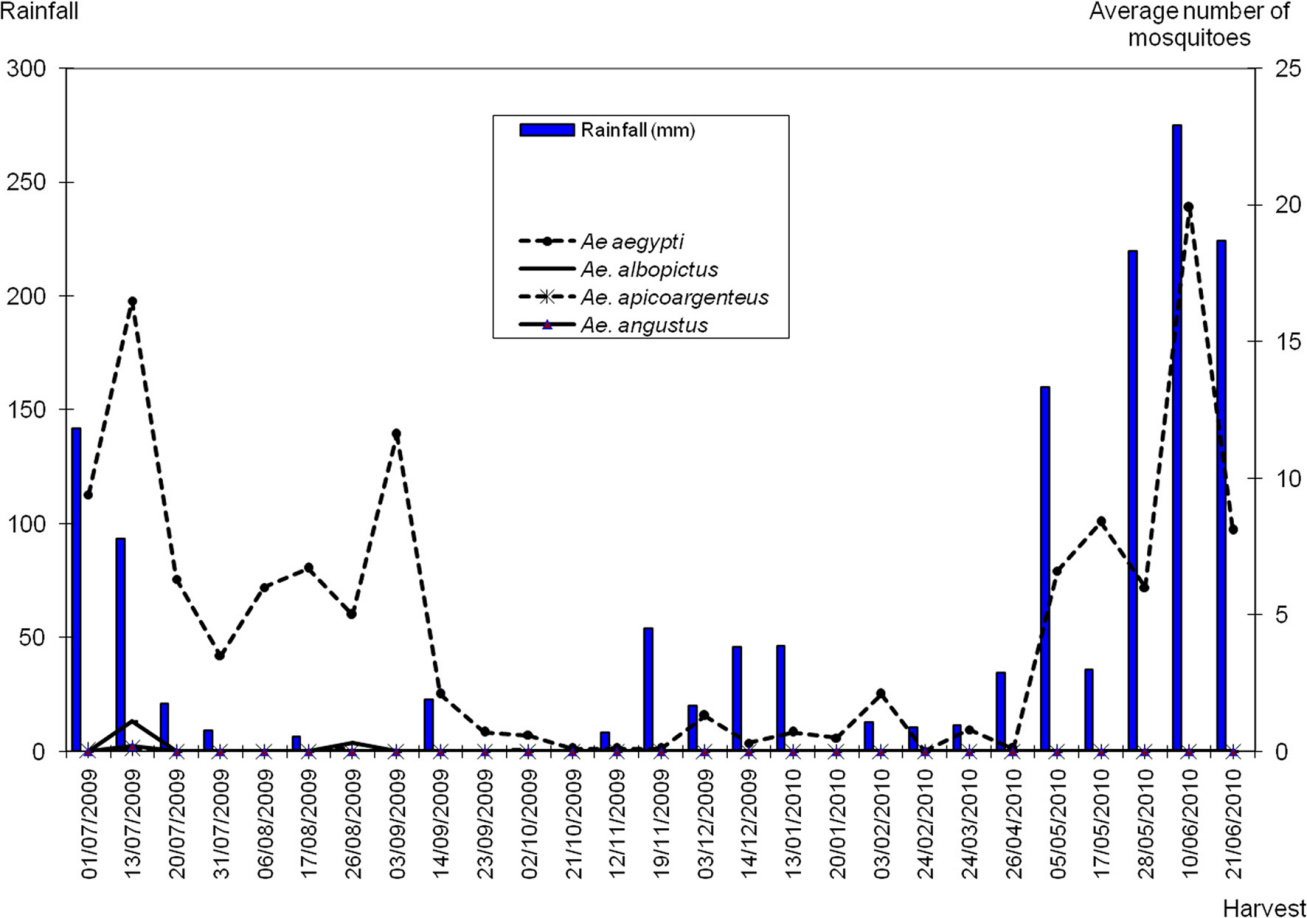
Average number of *Aedes* mosquitoes at the container terminal of the Autonomous Port of Abidjan (Côte d’Ivoire) from July 2009 to June 2010, in relation to rainfall.

The average number of *Aedes aegypti* obtained was 4.5 S/Pl. Its density variation was closely related to the amount of rainfall, with the density increasing or decreasing according to the abundance of rain. The highest densities were obtained respectively in July 2009 and June 2010 during the major rainy season. The lowest densities were obtained at the end of September 2009 and early October 2009. The effect of rain was clearly demonstrated again in the month of September 2009 where a peak density of 11.6 S/PL was obtained during the minor dry season because of 22.6 mm of rain recorded in one day. *Ae. albopictus* was collected at the end of the long rainy season with a maximum of 1.1 S/Pl in July 2009. *Ae. apicoargenteus* and *Ae. angustus* were collected only in July 2009, but in very low proportions (density ≤ 0.2 S/Pl) (Figure 1).

## Discussion

Data collected in this study helped to make the inventory of *Aedes* species present at the container terminal of the Autonomous Port of Abidjan while providing information about the population dynamics of *Aedes* with respect to the rainy season.

Despite the selectivity of ovitraps [23], *Culex* larvae were found in the water contained in cans of the ovitraps, at Empty park in February 2010. The *Aedes* species were composed of the two urban species (*Ae. aegypti* and *Ae. albopictus*) and two wild species (*Ae*. *apicoargenteus* and *Ae. angustus*). *Ae. aegypti* was the dominant species with 95.4% of the emergences. In addition, this mosquito was present throughout the study with nevertheless a low density of 0.1 specimen per paddle in April 2010 in contrast with the other three species that were obtained during the major rainy season. The abundance of *Ae. aegypti* can be explained by the multiplicity of its breeding sites resulting from human activity. Various breeding sites including empty cans and other discarded containers, bins of retention of residual water of air conditioners, safety helmets, tarpaulins, etc., favorable to the proliferation of mosquitoes were encountered at the container terminal. In the rainy season, they become functional breeding sites and thereby increase the population of this mosquito.

*Ae. albopictus,* whose presence was detected for the first time in Côte d’Ivoire, was probably introduced via containers or other imported goods from infested areas. It is a mosquito native to Southeast Asia which has infested all continents through the international trade of used tires [24, 25]. The female lays resistant eggs inside stored tires or other unprotected imported goods thereby enabling them to survive a long journey of several weeks. Apparently no serious attention is paid to hygienic conditions of imported goods so it is easy for these mosquitoes to arrive at the Container terminal. This clearly explains the presence of *Ae. albopictus* at Empty park, Cold park, and Workshop. Due to its opportunist feeding habit, this mosquito can be an excellent “relay” vector between other vertebrates and man. According to Rodhain [26], the expansion is very worrying to the extent that it could be the vector of a number of viruses and pathogens in many countries. In tropical areas, *Ae. albopictus* is known as a vector of dengue and other arboviruses including chikungunya virus, and of filariae [14, 18]. Moreover, transovarial transmission increases the potential threat that represents the introduction of this mosquito in Côte d’Ivoire where yellow fever and dengue viruses already circulate. *Ae. apicoargenteus* could probably come from the Banco forest where its presence has been highlighted with a maximum during rainy seasons [11] or probably from the coastal forests of Côte d’Ivoire [10]. That would explain its presence during the rainy season. In addition, it could also have been introduced at the port through both the vehicles transporting log for export and infested logs themselves. Logs containing holes can easily harbor eggs of this mosquito. This situation could also apply to *Ae. angustus* whose presence has not yet been mentioned in the Culicidae fauna of Côte d’Ivoire. *Ae. apicoargenteus* was collected in quantity in ovitraps but almost completely absent from the human bait catches, during an ecological study of the distribution and prevalence of potential vectors of yellow fever in the vicinity of Enugu in Nigeria [2]. Bauer [3] showed that this mosquito fails to become a host for yellow fever virus and, therefore, does not transmit the disease. Concerning *Ae. angustus*, very little information is so far available in the literature concerning the prevalence and distribution of this species in West Africa. This absence of information could also be due to a lack of interest on the species.

*Ae. aegypti* and *Ae. albopictus* are two known vectors in the transmission of arboviruses, including yellow fever, dengue, and chikungunya. The first two are diseases whose presences require obligatory declaration to WHO. These are diseases responsible for fatal epidemics which up to date have no specific cure. *Ae. albopictus* can be imported uninfected or infected. In the latter situation, the risk is more important even if it is necessary that the vector density be sufficient and that an important number of people be infected before an epidemic occurs. The monitoring of this species becomes important as it could introduce a cycle of dengue virus 1, 2, and 3 and chikungunya that already circulate in Côte d’Ivoire. It is therefore important to determine the origin of this mosquito, its potential expansion in the city of Abidjan, and its role in the emergence of future epidemics. The variation of the densities of *Aedes* mosquitoes observed indicates that epidemic risk is permanent. It is high at the end of the rainy season due to the presence of an abundant population of *Ae, aegypti* and the proliferation of other species of *Aedes* during the rainy season.

## Conclusion

This study revealed the presence of four species of *Aedes* at the container terminal of the Port of Abidjan, including two urban species and two wild species. *Aedes aegypti* was the dominant species and present throughout the study although collected in small proportion during the dry season. On the other hand, the other species were collected during the rainy season. This study also revealed the presence of *Ae. albopictus* and *Ae. angustus* in Côte d’Ivoire for the first time. It suggests the establishment of an entomological surveillance of potential vectors of arboviruses in the district of Abidjan. The port, the airport, and the Banco National Park should normally be pilot surveillance sites for a better management of these mosquitoes and their diseases.

## References

[1] Akoua-Koffi C, Ekra KD, Koné AB, Dagnan NS, Akran V, Kouadio KL, Loukou YG, Odehouri K, Tagliante-Aracino J, Ehouman A. 2002 Détection et gestion de l’épidémie de fièvre jaune en Côte d’Ivoire, 2001. Médecine Tropicale, 62(3), 305–309 12244930

[2] Bang YH, Bown DN, Onwubiko AO, Lambrecht FL. 1979 Prevalence of potential vectors of yellow fever in the vicinity of Enugu, Nigeria. Cahier ORSTOM, Série Entomologie Médicale et Parasitologie, vol. 3, 139–147

[3] Bauer JH. 1928 The transmission of yellow fever by mosquitoes other than *Aedes aegypti*. American Journal of Tropical Medicine, S 1–8(4), 261–282

[4] Brou Y. 1997 Analyse et dynamique de la pluviométrie en milieu forestier ivoirien. Thèse de Doctorat 3ème cycle, Université d’Abidjan, 200 p.

[5] Calder L, Laird M. 1994 Mosquito travelers, arbovirus vectors and the used tire trade. Travel Medicine International, 12, 3–12

[6] Crawley MJ. 2005 Statistics – an introduction using R. Wiley, 342 p.

[7] Condon R, Taleo G, Stewart T, Sweeney T, Kiedrzynski T. 2000 Surveillance de la dengue en Océanie. Pacific Health Dialog, 7(2), 131–136 11588914

[8] Cordellier R, Geoffroy B. 1972 Contribution à l’étude des vecteurs potentiels de fièvre jaune en République Centrafricaine, Cahier ORSTOM, Série Entomologie Médicale et Parasitologie, vol. 10, 127–145

[9] Deparis X, Maréchal V, Matheus S. 2009 Mécanismes physiopathologiques de la dengue : revue critique des hypothèses. Médecine Tropicale, 69(4), 351–357 19725385

[10] Doucet J, Adam J-P, Binson G. 1960 Les culicidés de la Côte d’Ivoire. Annales de Parasitologie Humaine et Comparée, 35(3), 390–408 13724040

[11] Doucet J, Cachan P. 1962 Moustiques forestiers de la République de Côte d’Ivoire. VI Observations sur les gîtes de ponte des moustiques du genre *Aedes* Meigen dans les arbres de la forêt du Banco (Abidjan). Bulletin de la Société de Pathologie Exotique, 55(3), 422–443

[12] Durand JR, Chantraine JM. 1982 L’environnement climatique des lagunes ivoiriennes. Revue d’Hydrobiologie Tropicale, 15(2), 85–113

[13] Edwards FW. 1941 Mosquitoes of ethiopian region. III: Culicidae adultes and pupae. London: London British Museum (Natural History), 449 p.

[14] Guillet P, Nathan M. 1999 *Aedes albopictus*, une menace pour la France? Médecine Tropicale, 59, 49–52 10549023

[15] Huang Y-M. 2004 The subgenus *Stegomyia* of *Aedes* in the Afrotropical Region with keys to the species (Diptera: Culicidae). Zootaxa, 700, 1–120

[16] Huynh Do P, Caumes E, Bricaire F. 2000 Les maladies infectieuses émergentes et réémergentes, un défi pour la santé publique. Praxis, 89, 125–132 10686805

[17] Konan YL, Koné AB, Ekra KD, Doannio JMC, Odéhouri-Koudou P. 2009 Investigation entomologique à la suite de la réémergence de la fièvre jaune en 2008 à Abidjan (Côte d’Ivoire). Parasite, 16, 149–152 19585894 10.1051/parasite/2009162149

[18] Mitchell CJ. 1995 Geographic spread of *Aedes albopictus* and potential for involvement in arbovirus cycles in the Mediterranean basin. Journal of Vector Ecology, 20, 44–58

[19] OMS 2003 Lutte contre la fièvre jaune en Afrique : progrès, contraintes et défis. Bulletin des Maladies Évitables par la Vaccination, OMS/AFRO, 31, 1–2

[20] OMS 2003 Yellow fever vaccine. Weekly Epidemiological Record, 78(40), 349–35914569711

[21] OMS 2009 La dengue en Afrique : émergence du DENV-3, Côte d’Ivoire, 2008. Relevé Épidémiologique Hebdomadaire, 84, 85–95 19280730

[22] OMS 2012 Dengue et dengue hémorragique. Aide-mémoire No. 117, Janvier 2012

[23] Pichon G, Gayral P. 1970 Dynamique des populations d’*Aedes aegypti* dans trois villages de savane d’Afrique de l’ouest : fluctuation saisonnière et incidence épidémiologique. Cahier ORSTOM, Série Entomologie Médicale et Parasitologie, VIII(1), 49–68

[24] Reiter P. 1988 Aedes albopictus and the world trade in used tires, 1988–1995: the shape of things to come? Journal of the American Mosquito Control Association, 14, 83–94 9599329

[25] Reiter P, Sprenger D. 1987 The used tire trade: a mechanism for the worldwide dispersal of container breeding mosquitoes. Journal of the American Mosquito Control Association, 3, 494–501 2904963

[26] Rodhain F. 1996 Problèmes posés par l’expansion d’*Aedes albopictus*. Bulletin de la Société de Pathologie Exotique, 89, 137–141 8924772

[27] WHO 2006 Scientific Working Group report on dengue. Meeting report 1–5 October 2006 Geneva, Switzerland. Available from: http://www.who.int/tdr/publications/documents/swg_dengue_2.pdf (consulted 12-03-2013)

